# A support vector machine model provides an accurate transcript-level-based diagnostic for major depressive disorder

**DOI:** 10.1038/tp.2016.198

**Published:** 2016-10-25

**Authors:** J S Yu, A Y Xue, E E Redei, N Bagheri

**Affiliations:** 1 Chemical and Biological Engineering, McCormick School of Engineering, Northwestern University, Evanston, IL, USA; 2 Department of Psychiatry and Behavioral Sciences, Feinberg School of Medicine, Northwestern University, Evanston, IL, USA

## Abstract

Major depressive disorder (MDD) is a critical cause of morbidity and disability with an economic cost of hundreds of billions of dollars each year, necessitating more effective treatment strategies and novel approaches to translational research. A notable barrier in addressing this public health threat involves reliable identification of the disorder, as many affected individuals remain undiagnosed or misdiagnosed. An objective blood-based diagnostic test using transcript levels of a panel of markers would provide an invaluable tool for MDD as the infrastructure—including equipment, trained personnel, billing, and governmental approval—for similar tests is well established in clinics worldwide. Here we present a supervised classification model utilizing support vector machines (SVMs) for the analysis of transcriptomic data readily obtained from a peripheral blood specimen. The model was trained on data from subjects with MDD (*n*=32) and age- and gender-matched controls (*n*=32). This SVM model provides a cross-validated sensitivity and specificity of 90.6% for the diagnosis of MDD using a panel of 10 transcripts. We applied a logistic equation on the SVM model and quantified a likelihood of depression score. This score gives the probability of a MDD diagnosis and allows the tuning of specificity and sensitivity for individual patients to bring personalized medicine closer in psychiatry.

## Introduction

Undiagnosed cases of major depressive disorder (MDD) pose a major detriment to society by contributing to disability,^1^ comorbid health conditions,^2^ and, in many cases, suicide.^3^ Approximately 16.2% of the US population suffers from MDD at least once in a lifetime,^2^ with significant percentages left undiagnosed, misdiagnosed, and/or untreated.^4^ Individuals with MDD are identified by self-reported changes in behavior, mood, and clinical examination.^5^ However, certain subpopulations—such as children,^6^ adolescents,^7^ and elderly individuals^8^—prove difficult to diagnose with these methods. This difficulty may be attributed to comorbid mood disorders, trouble in communicating with doctors,^9^ and unwillingness to seek clinical help because of social stigma.^10^ Alternatively, the subjective clinical screening tools employed for making the diagnosis—such as the Hamilton Depression Rating Scale, Beck Depression Inventory, or Patient Health Questionnaire-9—and the observed heterogeneity in *Diagnostic and Statistical Manual of Mental Disorders, Fifth Edition* symptom profiles^11^ call into question the reliability of diagnosing depression using subjective, symptom-based methods.

In addition, most depression is treated in primary care; it is estimated that 12.5% of primary care patients have had MDD in any given year, but only 47% of those cases are recognized clinically.^12^ Undiagnosed cases of depression can lead to a variety of social, economic, and emotional problems^1^ and the longer the diagnostic delay (estimated at 2–40 months), the more difficult it is to treat depression.^13^ The health and safety of depressed and non-depressed individuals can be compromised if mood disorders are not recognized and treated.^1^ Thus, an accurate and objective diagnostic method would be of great benefit to doctors, patients, and society as a whole.

Although depression is clearly a heterogeneous disorder, identifying biomarkers indicative of the most common symptom profiles^11^ is a road toward objectifying a subjective diagnosis. Finding molecular biomarkers in the blood provides a particularly useful source of information, as blood can be easily isolated and assayed in most clinical laboratories. In previous studies, Redei and colleagues discovered and measured the levels of 20 blood transcriptomic markers proposed to be associated with MDD: *CMAS, MARCKS, ATP11C, CDR2, CD59, CADM1, AMFR, FAM46A, DGKA, MAF, NAGA, RAPH1 PTP4A3, TLR7, ADCY3, ASAH1, ZNF291, PSME1, KIAA1539* and *SLC4A1*.^14, 15^ These transcriptomic markers were first identified in the blood of two etiologically differing animal models of depression by genome-wide transcriptomic analyses.^14, 16^ The unique blood and brain genome-wide expression profiles of the genetic depression model compared with its genetically close control strain were analyzed in conjunction with another study employing four different strains of rats exposed to chronic restraint stress versus non-stressed controls. Based on the differentially expressed genes in these two studies, blood transcriptomic markers where selected. The marker selection criteria included (i) simultaneous differential expression of markers in the blood and the brain in the genetic model, (ii) simultaneous differential expression of genes in the blood of all of the strains in the stress model, and (iii) conservation of the marker expression between the rat models and humans.^17^

Using the transcript levels measured for all 20 markers in the 64 subjects, three supervised machine-learning classification models—logistic regression,^18^ random forests,^19^ and support vector machines (SVMs)^20, 21^—were built to identify a subset of these markers that are predictive of MDD diagnosis. The utility and accuracy of these models were evaluated based on their ability to accurately distinguish subjects with MDD from the control no-disorder (ND) group. These models are commonly used in the bioinformatics and systems biology fields to predict certain phenotypic outcomes in large biological data sets.^22^ We refer readers to Ressom *et al.*^22^ for a more comprehensive summary of machine-learning models used to make diagnostic predictions with DNA microarray and protein mass spectrometry data.

This study integrates supervised machine learning with transcript-level data to identify features of RNA expression in peripheral blood that are predictive of MDD. We found that a SVM model with a linear classification boundary and a logistic probability distribution function offers high accuracy in identifying subjects with MDD. In addition to classifying MDD versus the ND control group, our model also provides a quantitative score that predicts the probability of a subject having MDD. We termed this probabilistic prediction the likelihood of depression (LiD) score. The high sensitivity (true-positive rate) and high specificity (true-negative rate) of our resulting model lay the foundation for a revolutionary, subjective, blood RNA expression-based diagnostic test for MDD. Such a test could improve the statistical significance of clinical trials for antidepressant treatments, enhance the rate of market approval and relieve the suffering for the many individuals who are adversely affected by undiagnosed depressive disorder.

## Materials and methods

### Clinical diagnosis and RNA expression profiles are collected from MDD and control subjects

Subjects were recruited as described.^15^ Subjects met criteria for MDD based on the Mini International Neuropsychiatry Interview^23^ and had a Hamilton Depression Rating Scale^24^ score ⩾16. Depression severity was evaluated by self-report using the Patient Health Questionnaire-9 (PHQ-9) for all subjects. No-disorder (ND) controls matched by age, race, and sex were included if they did not meet criteria for depression and scored ⩽4 on PHQ-9.

Venous blood (2.5 ml) was collected into PAXgene Blood RNA tubes (Qiagen, Germantown, MD, USA) from 32 subjects with MDD and from 32 ND controls. Blood RNA was extracted using the PAXgene Blood RNA Kit (Qiagen), according to the manufacturer protocol. The yield and quality of extracted RNA were assessed using the NanoDrop 1000 spectrophotometer (NanoDrop Technologies, Wilmington, DE, USA). cDNA was prepared using random primers and the TaqMan RT reagents (Applied Biosystems, Foster City, CA, USA). Expression levels of the 20 markers for each of the 64 subjects were measured using the quantitative real-time polymerase chain reaction (qPCR) method as described previously.^14, 16^ qPCR was carried out using SYBR Green and the ABI 7900 (Applied Biosystems), with 18s rRNA as the internal control and primers as published.^15^ The ΔCT values from qPCR characterized transcript abundance, where ΔCT is the cycle threshold difference between the target gene and the housekeeping gene.

### Supervised machine learning classifies MDD and control subjects

A supervised machine-learning model is a type of classification model that uses an explanatory variable, ***x***, to predict a known response, ***y***. Supervised models are trained on a subset of the known data and the corresponding predictive accuracy is assessed using held-out validation, or test, data. This process is known as cross-validation. Accuracy is defined as the percentage of correctly classified samples. In the matrix form, the explanatory variables are defined as ***X***, a measure of the abundance of RNA transcripts of specific genes from whole blood, and the response variables as ***y***, a set of binary values corresponding to the diagnosis of the subject. In this study, the binary diagnostic categories are subjects with MDD, Class 1, or subjects in the control group, Class 0. The general form of the predictive model is ***y=**f*(***X***;***p***), where the function *f* defines a mapping of the input (RNA expression) values to a response (MDD diagnosis) based on specified model parameters, ***p***.

The following three predictive models were used: logistic regression,^18^ random forests,^19^ and SVMs.^20, 21^ All models were implemented in Matlab 2016a (Mathworks, Natick, MA, USA). Logistic regression was built using the mnrfit function and default parameters. Random Forests was built using the TreeBagger function with 100 trees and otherwise default parameters. SVM models were implemented using either svmtrain or fitcsvm with settings to match svmtrain defaults (i.e., solver = Sequential Minimal Optimization, cache size = 5000). The SVM models were tested using linear, quadratic, polynomial, and radial kernel functions. Leave-one-out cross-validation was performed by omitting each of the 64 samples sequentially, training on the remaining 63 samples, and testing model prediction performance on the one left-out sample.^25, 26^

### Backward selection improves predictive power and identifies optimal variable set

Backward selection iteratively removes the least predictive features and regenerates new models to improve prediction.^26, 27^ The least predictive variable is identified by removing each variable and retraining a model on the reduced data set. The variable resulting in the smallest decrease in classification accuracy is considered the least predictive and permanently removed, and the process is repeated until a single feature remains. Backward selection was used for all three machine-learning methods to identify the feature set resulting in the highest predictive accuracy.^26, 27^

### Gene ontology analysis identifies molecular functions associated with the most predictive features

The eight genes found in the optimal model in both logistic regression and the linear SVM were defined as highly predictive. Enrichr was used to identify molecular functions enriched in these highly predictive genes.^28^ These functions were ordered by the negative log_10_ of the adjusted *P*-value quantifying the probability that a function was associated with the list of eight genes.

## Results

The samples in this study consisted of 32 subjects with MDD and 32 age- and gender-matched ND controls.^15^ We employed supervised machine-learning techniques to predict subject MDD diagnosis. These classification models were independently optimized and compared based on classification accuracy, specificity, and sensitivity. With the many explanatory variables common in large-scale genomic studies, models often “overfit” the parameters to the training set, leading to perfect classification of training data and poor classification of validation data. Therefore, a model with a high accuracy on training data can still suffer from poor predictive power. For this reason, we use a cross-validated accuracy to quantify predictive power. We chose leave-one-out cross-validation, where a single sample is removed and the model is trained on the remaining samples. The classification of the left-out sample is then predicted. Cross-validated accuracy is defined as the sum of the number of correctly classified left-out samples (true-positives and true-negatives) divided by the total number of samples.

We tested a number of predictive models that have previously been used successfully with similar data sets^29^ and compared their relative performance. The purpose of model fitting and analysis is to identify a model (or set of models) that can accurately predict whether a subject suffers from MDD based on a simple blood sample. For clinical relevance and impact, the model must also ensure both high sensitivity and high specificity. Sensitivity, or the true-positive rate, quantifies the proportion of subjects with MDD who are correctly identified; specificity, or the true-negative rate, quantifies the proportion of ND control subjects who are correctly identified.^26, 27^

### Logistic regression accurately classifies subjects with MDD

Classification models are often developed based on the assumption that a linear relationship exists between explanatory variables and responses. This assumption is formalized by the following equation:


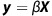


where *β* is a vector containing coefficients that represent the linear relation between explanatory variables ***X*** and response ***y***. For a response with two classes, the predicted response will be assigned to Class 1 if ***y*** is above a set threshold, and assigned to Class 0 if ***y*** is below the threshold. The function, *β**X***, forms a hyperplane, or boundary, that separates the two classes of responses. Logistic regression assigns a relationship between the explanatory variables and the response using the logistic equation:


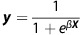


The logistic curve has an S-shape with the inflection point of the curve at the inferred boundary between the two classes. This model has the advantages of being both highly interpretable and easy to implement. In addition, logistic regression quantifies a probability that a sample exists in a given class, which is a function of the distance of the sample from the inferred boundary. For example, a subject who expressed genes that fall near the boundary would have a 50% probability of being in Class 0 or Class 1. A subject whose transcriptomic panel falls far from the boundary may have a probability of 99% of belonging to Class 1 and a 1% probability for belonging in Class 0. We applied logistic regression to predict the probability of a new subject belonging to the MDD class, given the expression of blood transcript levels of the 20 genes.^14, 15^

### Backward selection increases predictive power with fewer transcript variables

**Figure 1:**
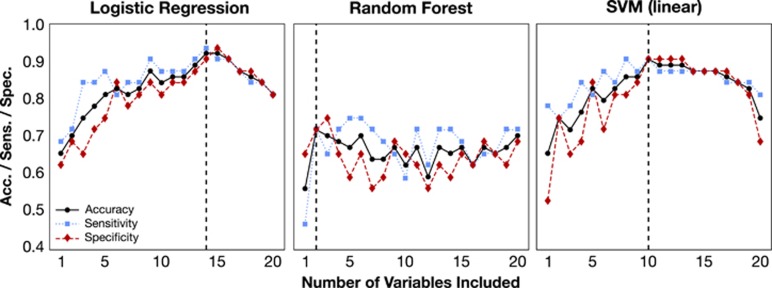
A linear boundary SVM and logistic regression outperform random forests in identifying subjects with MDD. Three supervised machine-learning methods were applied to discriminate MDD subjects from control subjects: (left) logistic regression, (center) random forests, and (right) support vector machines. To improve model prediction and identify an optimal transcript set, backward selection was performed. Backward selection removes transcripts from the explanatory variables in the classification model individually; for each iteration, we recalculate model accuracy, sensitivity, and specificity. The transcript associated with the lowest accuracy is permanently removed from the set of predictive variables and the process is repeated. Random forests had less accuracy than logistic regression or SVMs, suggesting that nonlinear contributions of the explanatory variables did not provide additional accuracy to the model. Logistic regression and SVMs with a linear boundary both had high accuracy, 92.2% and 90.6%, respectively. MDD, major depressive disorder; SVM, support vector machine.

We employed backward selection to fit subject diagnosis of increasingly smaller transcript sets and calculated cross-validated accuracy, sensitivity, and specificity for each selection step (Figure 1, left). The logistic regression model exhibited maximum accuracy with the inclusion of 14 transcript variables, suggesting that this subset of transcripts has the most predictive ability. The 14 genes are *RAPH1*, *CDR2*, *CMAS*, *DGKA*, *AMFR*, *NAGA*, *ZNF291*, *PSME1*, *TLR7*, *PTP4A3*, *ATP11C*, *CADM1*, *MAF*, and *ASAH1*. The 14-variable model had a high classification accuracy of 92.2%, demonstrating that the data had significant power to accurately predict MDD in subjects outside of the training data. In addition, the model had a high cross-validated sensitivity and specificity of 93.8% and 90.6%, respectively.

Although logistic regression yielded a highly accurate and interpretable result, it assumes that there is a linear relationship between the input variables and the output. We therefore inquired whether nonlinear effects can improve the accuracy of the model. We applied random forests, which allow for nonlinear relationships between transcripts.^16^

### A random forests classification model predicts MDD with moderate accuracy

Random forests uses an ensemble of decision trees to classify a response variable based on a matrix of explanatory variables.^19^ Random forests perform well on diverse data sets as it makes few assumptions on the structure of the data; it does not require a linear relationship between the input variables and the response. The model minimizes overfitting by taking random subsets of data samples and explanatory variables and aggregating the predicted responses in a process known as Bootstrap Aggregating.^19^ The predicted class of the response is the majority vote over all of the bootstrap-aggregated trees.

In this study, we applied random forests to classify MDD subjects from controls. As random forests can create nonlinear relations between input variables and responses, the order of variable importance had little overlap with the logistic regression model. This difference may reveal nonlinear relationships between transcripts, which include synergistic or antagonistic contributions of multiple explanatory variables. In order to investigate this hypothesis, we compared the accuracy of random forests to logistic regression. Backward selection was repeated iteratively (Figure 1, center). Random forests performed worse than logistic regression in all 20 backward selection steps, suggesting that the nonlinear relationships were not meaningful or underpowered and did not add additional predictive power to the model.

### SVMs offer similar accuracy to logistic regression using fewer transcript variables

To further improve upon the predictive accuracy of our classification model, we implemented a third model, known as SVMs.^29, 30^ SVMs infer a multidimensional decision boundary that separates classes of variables. Similar to logistic regression, SVMs are interpretable; the multidimensional boundary can be visualized as projections on two-dimensional coordinates. SVMs also require less computational power than both random forests and logistic regression by using only the data points, termed support vectors, that define the boundary between classes. This computational strategy allows the model to be less sensitive to outliers than logistic regression, as the model is built only using samples close to the classification boundary.

SVMs may identify linear or nonlinear relationships depending on the type of boundary that is used to separate subject classes. A common boundary is an *n*−1 dimensional hyperplane that separates classes in an *n-*dimensional space. This type of classification boundary is linear as the hyperplane collapses to a line when projected into 2D space. Backward selection was applied to improve predictive power and identify predictive variables (Figure 1, right). SVMs had an optimal accuracy with the following 10 transcript abundances: *DGKA, CDR2, PSME1, ZNF291, AMFR, RAPH1, CMAS, NAGA, CD59*, and *SLC4A1.* The linear SVM model had a classification accuracy of 90.6% with 10 transcript variables, four fewer variables than logistic regression. This result corresponds to a desirable sensitivity and specificity of 90.6% and 90.6%, respectively, suggesting that the linear SVM model may be most promising for clinical application. Other SVM variations employing nonlinear boundaries^30^ are described in Supplementary Figure 1. The linear boundary outperformed all nonlinear boundaries tested, further suggesting that the assumption of linearity is valid and that a linear relationship truly exists between the levels of specific RNA transcripts in the blood and MDD diagnosis.

### SVMs can separate subjects with MDD from controls with high classification accuracy

**Figure 2:**
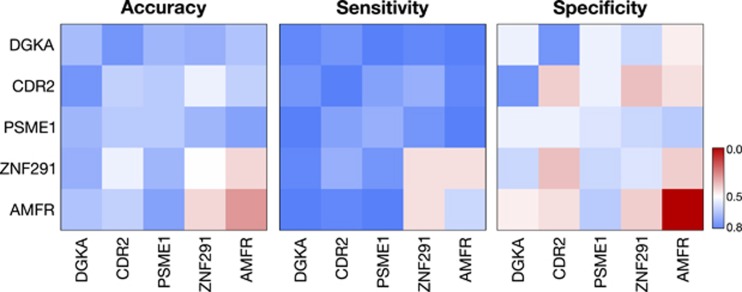
Combinations of RNA measurements have high predictive power, though individual measurements can be non-predictive. The heat maps contain the cross-validated accuracy, specificity, and sensitivity of pairwise combinations of the top five predictive transcripts from backward selection with linear SVMs. Even though *AMFR* had high predictive value for classifying MDD in conjunction with other variables, it had no predictive power on its own, as demonstrated by a specificity of 0%. Combinations of transcripts can inform a useful SVM boundary even if single transcripts have no ability in isolation, suggesting that depression is associated with combinations of genes. Note that backward selection does not comprehensively explore all transcript combinations and the complex relationships among transcripts suggest that a more predictive combination may still remain. MDD, major depressive disorder; SVM, support vector machine.

To further explore the predictive power of the SVM model, the five most important explanatory variables, as determined by the final five variables in backward selection, were used pairwise to build SVMs. The resulting boundaries separating MDD from controls are illustrated in Supplementary Figure 2. Accuracy, sensitivity, and specificity for each pairwise combination of these top five transcript variables are shown in Figure 2. Single transcripts, such as *DGKA* and *CDR2*, have predictive power in classifying depressed subjects without being combined with other explanatory variables. Conversely, *AMFR* and *ZNF291* were found to be important when in combination with other transcripts, but had little predictive power in isolation. It is possible for an individually non-predictive variable to have high predictive power in combination.^31^ For example, *AMFR* has a specificity of 0%, meaning the SVM classified all subjects with MDD. However, both specificity and sensitivity increase in combination with *PSME1* (81.2 and 62.5%), which is higher than *PSME1* alone (68.8 and 56.2%). This difference suggests that depression diagnosis may not be associated with the expression of a single gene, but rather the expression dysregulation of a combination of genes. Whereas levels of transcript variable pairs could have significant informative value in classifying depression, backward selection demonstrated that 10 transcript abundances had the highest accuracy with a sensitivity and specificity of 90.6% and 90.6%, respectively. This result underlines the complexity of MDD, suggesting that the heterogeneous nature of the illness and its etiology may be reflected in the expression patterns of combinations of specific genes.

### SVMs with a logistic equation fit provide an LiD score: the probability that a subject has MDD

As both logistic regression and SVM classification models had high accuracies in predicting MDD, we hypothesized that a combination of these two methods may be more useful than either in isolation. The SVM model is more robust to outliers and has fewer explanatory variables; however, logistic regression is able to assign a quantitative probability of being correctly classified depending on the distance that a new data point falls from the inferred boundary. We therefore used SVMs to infer the boundary and then set the inflection point of the logistic equation to this boundary to form a quantitative score for the probability that a new subject has MDD.^21^

For interpretive purposes, we selected the pairwise combination of the expression levels of the genes *CDR2* and *DGKA* to demonstrate the relationship between the SVM boundary and the logistic contours.


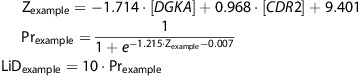


**Figure 3:**
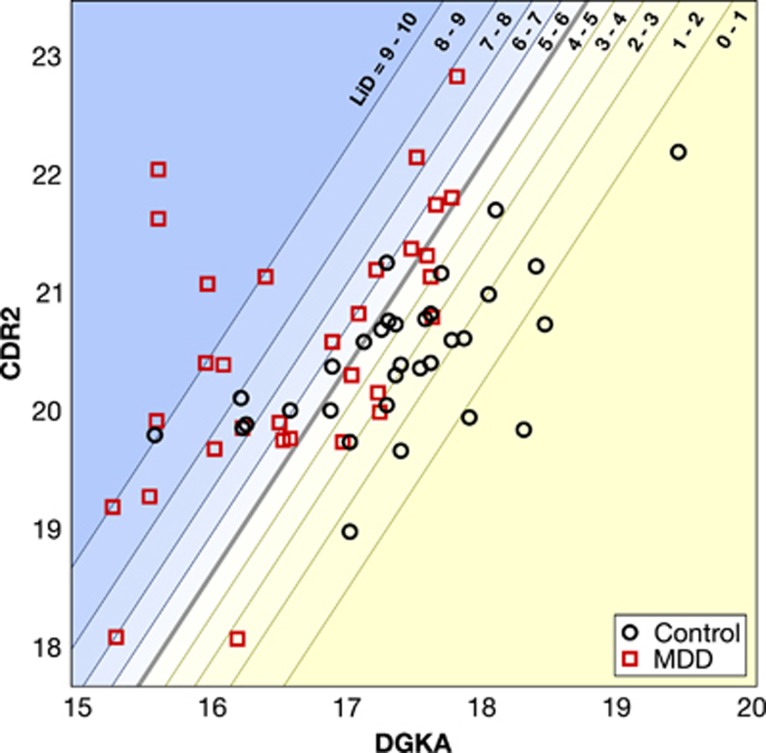
SVMs combined with a logistic equation provides quantitative LiD score corresponding to the probability of a MDD diagnosis. A logistic equation was fit on the boundary inferred by the linear SVM from the 10 most predictive transcripts. For illustrative purposes, we show the same method on the two most predictive pairwise genes, *DGKA* and *CDR2*; the full predictive model uses 10 transcripts and would be impractical to visualize. The thick line corresponds to the logistic regression inflection point and the thin lines correspond to deciles of probability of a MDD diagnosis fitted from logistic regression. The LiD score range is shown for each region. The overlaps in transcript measurements between MDD and ND control subjects highlights the inherent noise in MDD diagnoses as well as biological experiments. However, the probabilistic interpretation from logistic regression offers a diagnostic tool useful for clinicians. LiD, likelihood of depression; MDD, major depressive disorder; ND, no-disorder; SVM, support vector machine.

Z contains the weighted linear combination of the two RNA transcripts. This equation defines the hyperplane separating MDD from the control class. Pr is the probability of a subject having MDD as a function of the distance from the boundary calculated using a logistic equation. The LiD equation converts the probability to an interpretable number ranging from 0 to 10. This pair of transcripts had the highest accuracy in predicting MDD. Figure 3 shows a scatterplot of these two representative transcript variables in 2D space. The thicker boundary line in the figure shows the central boundary as determined using the SVM. The inflection point of the logistic equation was set at this boundary and the slope of the distribution was fit to the distribution of data surrounding the boundary. The probability of depression is predicted as a function of the transcripts' weighted distance from the SVM-defined boundary. This method allows the assignment of a quantitative score of 0 to 10 for each subject. We termed this score the LiD score, which quantifies the probability of having MDD.

The contours show the divisions between the deciles of probability of MDD. For example, subject samples that fall in the upper left part of the plot have a 90–100% probability of having MDD, whereas those that fall in the lower right have 0–10% probability. A subject falling on the central boundary would have a 50% chance of having MDD using this approach. As this figure demonstrates, the orthogonal distance from the central boundary can be mapped to a quantitative score for MDD using a logistic distribution. The LiD score can be useful for clinicians to determine, on a patient-by-patient basis, whether higher sensitivity or specificity is more important. The tolerance for false-positives and false-negatives may change depending on other clinical evidence, including medical comorbidities or treatments. This score may lead to a more informed, personalized diagnosis for MDD. At the 50% decision boundary, this combined model has a classification accuracy of 92.2%, sensitivity of 90.9% and specificity of 93.5%.

The LiD score ranges from 0 to 10 and corresponds directly to the percentage that a patient is in the MDD class as opposed to the ND control class. If the model predicts that a patient has a 50% probability of being in the MDD class, the corresponding LiD score is 5.0. The full model includes the 10 gene transcripts from the linear SVM with LiD equations:


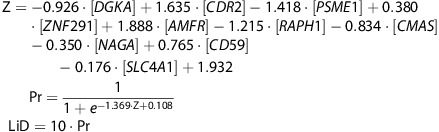


Similar to the example equations, Z describes the most predictive linear SVM model composed of the weighted linear combinations of 10 RNA transcripts; Pr is the probability of an MDD diagnosis, and LiD is an interpretable score ranging from 0 to 10.

Depending on prior knowledge, a clinician may select different tolerances for possible false-positives (misdiagnosis of MDD) and false-negatives (failure to diagnose MDD) for the patient. The corresponding sensitivities and specificities for different thresholds are given in Table 1. These values are for the full data set and are therefore slightly higher than the cross-validated sensitivities and sensitivities given in Figure 1. A threshold of 4 yields the highest sensitivity and specificity of 96.8% and 93.9%, respectively, and would be appropriate across a wide range of subjects with minimal false-positives or false-negatives. The performance of this model also suggests that a 10-transcript abundance sample from whole blood may be highly effective, widely adaptable, and easily scalable to a large subject population at risk for suffering from MDD.

### Predictive transcripts suggest mechanisms associated with MDD

**Figure 4:**
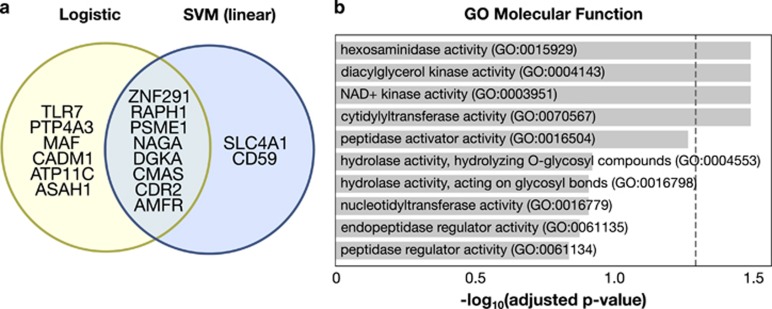
Some genes were highly predictive for diagnosing MDD, suggesting the biological processes underlying the etiology of major depression. Logistic regression and SVMs both identified genes with significant ability to predict MDD. (**a**) Maximum predictive power was achieved in logistic regression with 14 transcripts and in SVMs with 10 transcripts, with eight in common. These eight genes are hypothesized to have importance in explaining the biological processes underlying MDD. (**b**) Enrichr was used to find biological processes enriched in the eight common variables between logistic regression and SVMs. Four processes, as defined by the Gene Ontology (GO) database, were found to be significantly enriched (GO:0015929, GO:0004143, GO:0003951, and GO:0070567). The dotted line indicates the significance cutoff (adjusted *p*-value of 0.05). Together, these results suggest that multiple converging pathways may have independent roles in contributing to the depressive phenotype, and that MDD may have independent causal factors. MDD, major depressive disorder; SVM, support vector machine.

The most predictive variables may also give mechanistic insight into the etiology of depression. Both logistic regression and SVMs had a high overlap in the predictive variables. The most accurate SVM model consisted of 10 transcripts and the most accurate logistic regression model consisted of 14 transcripts, with eight transcripts in common between the two models (Figure 4a). We used these eight genes to infer molecular functions that may be associated with MDD.

*ZNF291*, *RAPH1*, *PSME1*, *NAGA*, *DGKA*, *CMAS*, *CDR2* and *AMFR* were found to be common significant explanatory variables between the two models. *TLR7*, *PTP4A3*, *MAF*, *CADM1*, *ATP11C* and *ASAH1* were selected solely by logistic regression. *SLC4A1* and *CD59* were unique to the SVM. The Enrichr suite^28^ was used to identify molecular functions, as defined by the Gene Ontology (GO) database, which may be associated with MDD. Four molecular functions were identified as significant (adjusted *P*-value<0.05) with these eight genes (Figure 4b): *hexosamidinase activity* (GO:0015929), *diacylglycerol kinase activity* (GO:0004143), *NAD+ kinase activity* (GO0003951) and *cytidylyltransferase activity* (GO:0070567). Notably, hexosamidinase deficiency occurs in late-onset Tay-Sachs disease,^32^ where some individuals experience psychiatric disturbances including depression and diacylglycerol kinase has been implicated in bipolar disorder.^33^ Cytidylyltransferases catalyze a rate-limiting step in the production of neural membrane glycerophospholipids, which are implicated in major depression and anxiety disorders.^34, 35^ It is of interest that all of these molecular functions are related to enzyme activity. It has been suggested previously that inborn or acquired errors of metabolism are often accompanied by psychiatric symptoms, whether as a consequence or a cause, only future research can tell.^36^ These molecular characterizations also suggest that there may be multiple converging causes that lead to depression through altering the availability of proteins, perhaps specifically enzymes, central to the etiology of MDD.

## Discussion

This is the first report to our knowledge that describes in complete detail different classification models for future diagnostic purposes built using a panel of transcript abundances. We compared several classification models to identify the most predictive model in discriminating subjects with and without MDD. Models were selected who had previously been shown to predict diagnostics in DNA microarray and protein mass spectrometry data.^22^ The logistic regression and SVM models offer high sensitivity and specificity in predicting subjects with MDD. Both models have their advantages: logistic regression can assign a probability of a subject having MDD and the SVM model has fewer explanatory variables and is less sensitive to outliers.

We therefore combined both methods to create an accurate and quantitative score to assign a probability of a patient having MDD from 10 biomarker measurements in the blood. This score, which we termed the LiD score, directly corresponds to the probability of a patient having MDD. Varying threshold can be applied for the LiD score depending on prior information about the patient or the risk involved in misdiagnosis. Given the subjective nature of MDD diagnosis, obtaining a true-positive (correct diagnosis of MDD) is likely more important than identifying a true-negative (correct diagnosis of no MDD), which suggests using a lower LiD threshold to improve sensitivity. In certain cases, such as with soldiers returning from active duty who have a high risk of having undiagnosed MDD and suicide,^37^ it may be appropriate to set the diagnosis threshold even lower to minimize the high cost of a false-negative. On the other hand, a higher threshold may be used with patients who have an existing relationship with a psychiatrist, minimizing the chance of a incorredt diagnosis of MDD leading to unnecessary therapy. A clinician's expertise can be combined with the LiD score to bring more accurate and personalized diagnosis to each patient. In addition, LiD score thresholds can be tuned in accordance with the distribution of MDD among a population.^38^ Because the non-MDD population is greater than the MDD population, the LiD score threshold can be raised to increase specificity and reduce false-positives.

A further advantage of the LiD score is its reproducibility and quantitative nature. The high prevalence of MDD motivates pharmaceutical companies to pursue effective treatments for MDD. The LiD score can be used as a quantitative measure of baseline and improvement in clinical trials. A small increase in the statistical significance of a change in efficacy among new drug candidates can be the deciding factor between a failed clinical trial and FDA approval for the launch of new life-saving medications.

The small number of gene transcripts suggests that not only is it possible to detect MDD from a blood sample alone, but also that such a test can be applied with low cost on a large scale. Blood RNA can be easily stabilized, stored, and transported without the need of complicated protocols or expensive refrigeration during transport. Blood draws and qPCR are already widely accessible in clinical settings and therefore the cost can be controlled across populations currently not equitably served by specialized psychiatric care.^39, 40^ The infrastructure for qPCR-based tests is already in place and would allow quick and widespread implementation to clinics across the country.

This RNA-based diagnostic has several advantages over other biomarker-based diagnostics for depression. qPCR is a highly sensitive and high-throughput method, allowing for rapid, reliable quantification of biomarker levels. Blood samples collected for RNA isolation could be transferred from the collection to the laboratory sites, which is particularly useful in geographic areas less served by psychiatric health-care providers. The use of cDNA standards for each of the transcript markers could eliminate the differences in qPCR results between the different laboratories, as is the case for any other clinical quality-controlled measurements. A commercially available test, using a combination of serum-based biomarkers, has been proposed to identify the probability of depression in a subject by giving an MDD score.^41^ The serum-based measures include alpha 1 antitrypsin, apolipoprotein CIII, myeloperoxidase, soluble tumor necrosis factor a receptor II, cortisol, epidermal growth factor, prolactin, resistin, and brain-derived neurotrophic factor.^41^ This study relies on protein biomarkers and specialized proprietary laboratories, where the MDD score is calculated. The algorithm for this MDD score is not publicly available. In addition, the protein-based study had a lower sensitivity and specificity (91.7 and 81.3%).^41^ We were able to achieve a higher sensitivity of 96.8% and a specificity of 93.9% at an LiD threshold of 4, suggesting a lower chance of misdiagnosis.

In addition to creating a blood-based diagnostic for depression, our approach also identified novel genes with expression differences in MDD that are either the consequence of, or are involved in, the etiology of MDD. Transcripts with the highest predictive power may be critical to the etiology of depression. Using the eight transcript variables that were in common in the most important variables for logistic regression and SVMs, we performed gene ontological analysis and identified four significant (adjusted *p*-value < 0.05) molecular functions. These functions are all related to enzymes, including several having shown relevance with mental health diseases: hexosaminidase and Tay-Sachs disease,^32^ diacylglycerol kinase and bipolar disorder,^33^ and cytidylyltransferases and neural membrane glycerophospholipids.^34, 35^ These cross-relations are unsurprising given the complex nature of mental health diseases and suggest that MDD may have multiple independent causes that can occur in isolation or combination in patients. Future large-scale studies using these markers together with the SVM classifier would greatly facilitate identifying the subtype of depression that best describes each patient, bringing treatment to a personal level.

An accurate and objective blood-based diagnostic test for depression has societal value by (i) decreasing the number of undiagnosed individuals who suffer from MDD, (ii) providing clinicians a quantitative probability of MDD diagnosis to inform personalized therapy, and (iii) improving the statistical significance of clinical trials for new antidepressant medications. We propose a SVM-based technique that offers highly accurate MDD diagnosis and the LiD score that corresponds to the probability that a patient has MDD. This study further suggests multiple, independent processes that may be involved in the etiology of depression, which could inform new research directions and possible drug targets. The success of SVMs in identifying subjects with MDD also demonstrates a computational means by which to identify subgroups of subjects that may respond to specific treatments, further facilitating the speed, ease, and accuracy with which doctors may treat patients with depression. In summary, the SVM-based LiD score derived from an RNA-based blood test may revolutionize the treatment of depression to greatly benefit pharmaceutical companies, clinicians, and ultimately the individuals suffering from undiagnosed MDD.

## Figures and Tables

**Table 1:** LiD thresholds can be tuned on a patient-by-patient basis

*LiD threshold*	*Accuracy*	*Sensitivity*	*Specificity*
>1	0.672	0.923	0.608
>2	0.812	0.955	0.738
>3	0.844	0.958	0.775
>4	0.953	0.968	0.939
>5	0.922	0.909	0.935
>6	0.875	0.816	0.962
>7	0.875	0.800	1.000
>8	0.719	0.640	1.000
>9	0.703	0.627	1.000

Abbreviation: LiD, likelihood of depression score.
