# Supplementary figures and images for: A support vector machine model provides an accurate transcript-level-based diagnostic for major depressive disorder
Source: Transl Psychiatry. 2016 Oct 25;6(10):e931–. doi: 10.1038/tp.2016.198 (PMC5290347; doi:10.1038/tp.2016.198)

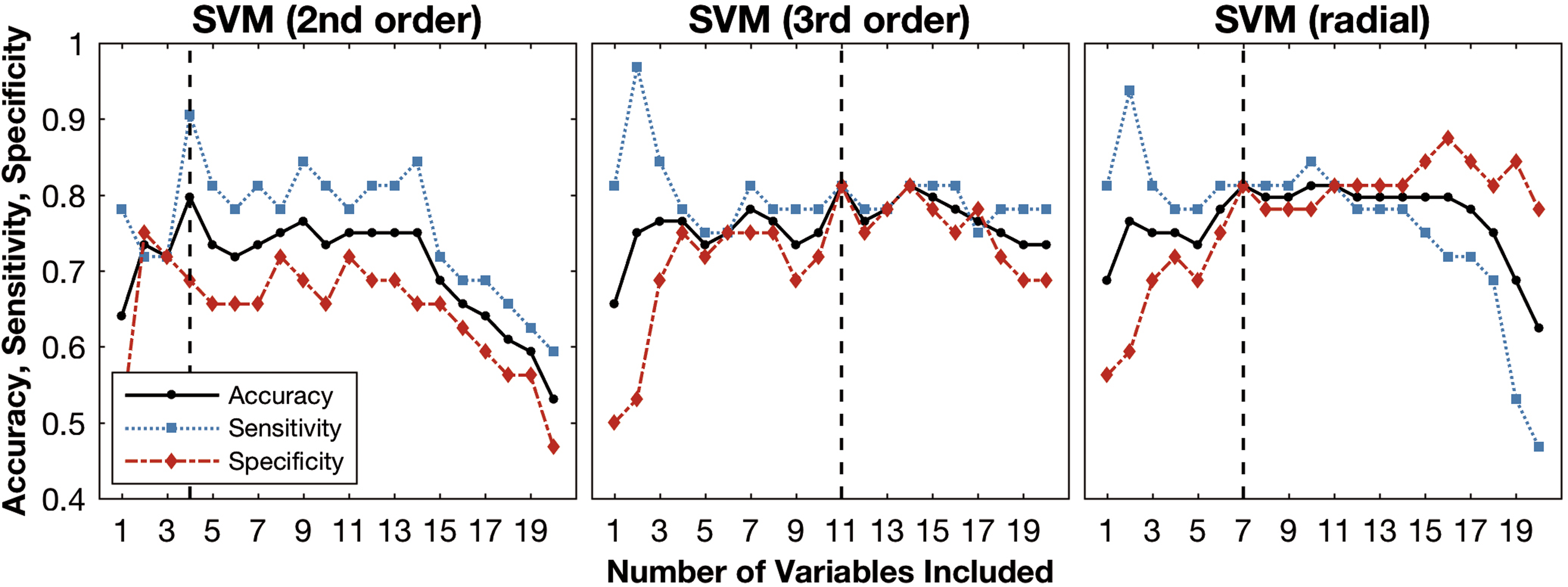

Supplement: Supplementary Figure 1 [file tp2016198x1.tif]

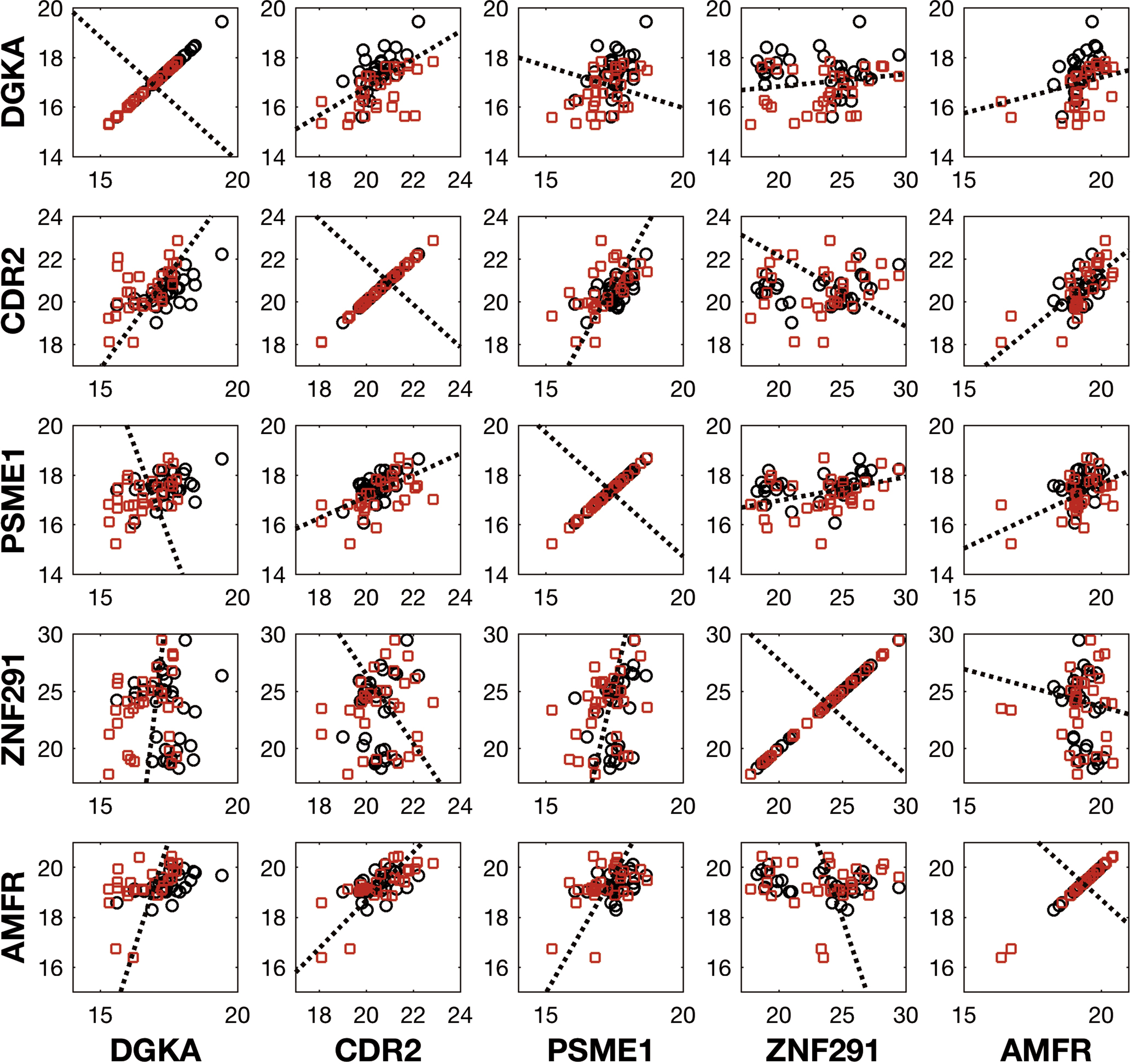

Supplement: Supplementary Figure 2 [file tp2016198x2.tif]
