# Supplementary material for: A support vector machine model provides an accurate transcript-level-based diagnostic for major depressive disorder
Source: Transl Psychiatry. 2016 Oct 25;6(10):e931–. doi: 10.1038/tp.2016.198 (PMC5290347; doi:10.1038/tp.2016.198)
Supplement: Supplementary Figure Legends [file tp2016198x3.docx]

# Supplementary Figure Legends

**Supplemental Figure 1. Nonlinear support vector machines do not perform as well as the linear support vector machine.** Three non-linear boundaries: (left) quadratic, (center) third-order polynomial, and (right) radial support vector machines were applied. Each plot shows model accuracy, sensitivity, and specificity across each round of backward selection. All three non-linear boundaries perform worse than the linear boundary, suggesting that a linear relationship exists between transcript abundance and MDD diagnosis.

**Supplemental Figure 2. Pairwise linear SVM using the top five most important features as determined using backward selection.** The dashed black line marks the SVM boundary. Control (black circles) and MDD (red squares) transcript abundances for the two genes in the pair are plotted.
